# Reaction Norms in Natural Conditions: How Does Metabolic Performance Respond to Weather Variations in a Small Endotherm Facing Cold Environments?

**DOI:** 10.1371/journal.pone.0113617

**Published:** 2014-11-26

**Authors:** Magali Petit, François Vézina

**Affiliations:** 1 Département de Biologie, chimie et géographie, Université du Québec à Rimouski, 300 Allée des Ursulines, Rimouski (Québec), G5L 3A1, Canada; 2 Groupe de recherche sur les environnements nordiques BOREAS, Rimouski (Québec), Canada; 3 Centre d'Etudes Nordiques, Québec (Québec), Canada; 4 Centre de la Science de la Biodiversité du Québec, Montréal (Québec), Canada; Universidad de Granada, Spain

## Abstract

Reaction norms reflect an organisms' capacity to adjust its phenotype to the environment and allows for identifying trait values associated with physiological limits. However, reaction norms of physiological parameters are mostly unknown for endotherms living in natural conditions. Black-capped chickadees (*Poecile atricapillus*) increase their metabolic performance during winter acclimatization and are thus good model to measure reaction norms in the wild. We repeatedly measured basal (BMR) and summit (Msum) metabolism in chickadees to characterize, for the first time in a free-living endotherm, reaction norms of these parameters across the natural range of weather variation. BMR varied between individuals and was weakly and negatively related to minimal temperature. Msum varied with minimal temperature following a Z-shape curve, increasing linearly between 24°C and −10°C, and changed with absolute humidity following a U-shape relationship. These results suggest that thermal exchanges with the environment have minimal effects on maintenance costs, which may be individual-dependent, while thermogenic capacity is responding to body heat loss. Our results suggest also that BMR and Msum respond to different and likely independent constraints.

## Introduction

Phenotypic flexibility is the ability of a fully-developed organism to rapidly and reversibly adjust its phenotype to track short-term environmental changes [1]. Because it allows for individuals to match their physiology with the requirements of their surroundings, phenotypic flexibility should improve survival and thus influence fitness [2], [3].

**Figure 1:**
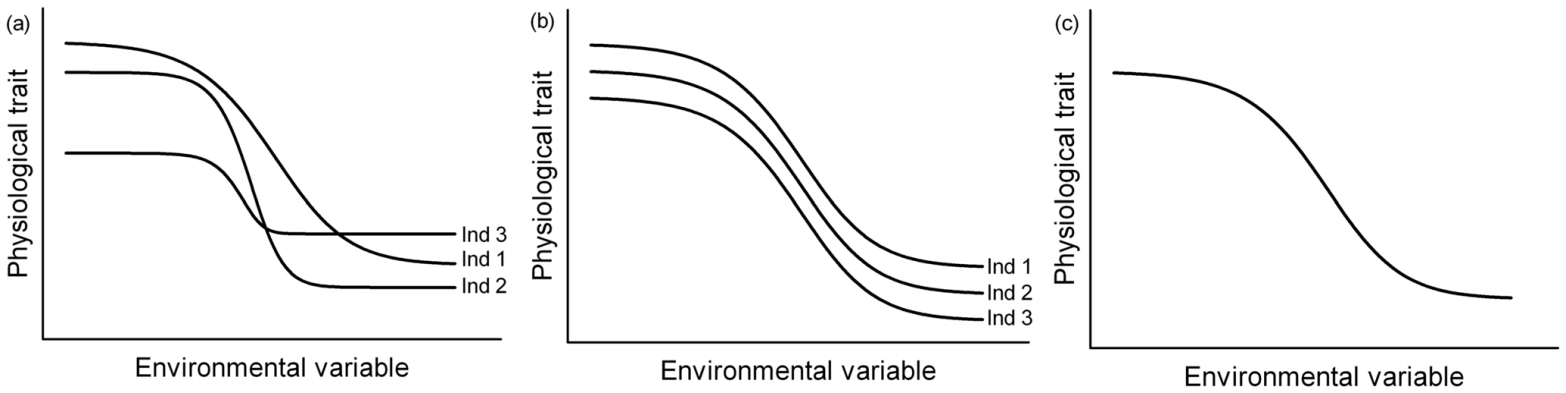
Reaction norms according to three scenarios. In (a) individuals express different elevations and slopes, in (b) individuals express different elevations but similar slopes and in (c) individuals express the same elevations and slopes, which reflect the population reaction norm.

The capacity to adjust phenotypic traits to changes in the environment is studied through reaction norms, which describes the flexibility of a trait across an environmental gradient (Figure 1) [4]–[6]. According to Nussey *et al.* 2007 [5], Brommer 2013 [3] and Mc Kechnie 2008 [6], reaction norms can be characterized by four parameters. The *elevation* is the mean trait expression (*i.e.* the intercept), the *slope*, which represents phenotypic flexibility, measures the change in trait value for a given change in an environmental parameter, the *amplitude* is the difference between minimal and maximal trait values and the *shape* (*e.g*. linear, sigmoid) informs on the limits of adjustment in a trait over a given range of change in the environment.

Reaction norms can be studied both at individual (Figure 1a and b) and population levels (Figure 1c) [3], [7]. In a first scenario (Figure 1a), individuals with different elevations and slopes show different phenotypes and phenotypic responses to changes in their environment. If phenotypic flexibility is under natural selection [8], environmental stochasticity should promote survival of the most flexible individuals, leading to microevolution at the population level [5]. In a second scenario (Figure 1b), animals with different elevations but similar slopes express different phenotypes but have the same flexibility. Individuals with the highest elevation would consistently remain high relative to others (*i.e.* repeatable trait) and, assuming that a high elevation in that trait increases fitness, they would perform better than their counterparts [9]. In a third scenario (Figure 1c), individuals express similar elevations and slopes resulting in individuals with comparable phenotypes and phenotypic flexibilities. In this situation, individual phenotypic flexibility would reflect the adjustment capacity of the population [5].

Animals facing highly variable environments, such as those wintering at high latitudes, offer a great opportunity for studying phenotypic adjustments in natural conditions. Indeed, winter is a challenging period for non-hibernating endotherms living at northern latitudes [10], [11] as they have to face low food availability [12] at a time where high energy expenditure is required for thermoregulation [13]. Winter metabolic phenotypes, reflecting individual rates of energy use, are commonly studied to understand individual performance and are measured through variables such as basal and summit metabolic rates (BMR and Msum, respectively). BMR is interpreted as the minimal maintenance energy cost of an animal and is thought to reflect metabolic activity of resting muscles and internal organs [14], [15] while Msum is a measure of the cold-induced maximal heat production that is thought to reflect maximal shivering capacity of skeletal muscles [16], [17]. In small endotherms, both these parameters are typically elevated in winter relative to summer [18], [19].

Adjustments in traits, such as metabolism, over a continuum of environmental variation are commonly investigated in ectotherms [20]. However, only few studies are available for endotherms, with the majority focussing solely on the effects of winter ambient temperature [21]–[23]. Studies on acclimatization typically report differences between seasonal extremes with metabolic rates being higher in winter than in summer [13], [19], although observations for periods shorter than 6 months are also available [19], [24]–[26]. Similarly, experimental studies investigating metabolic adjustments to thermal variations in endotherms typically use discrete changes [27], [28] rather than a continuous gradient of temperature. These approaches therefore limit conclusions to stable physiological states and provide little information on the dynamics of change in physiological parameters.

McKechnie [6] suggested that flexibility of metabolic rate could be limited by physiological or morphological constraints, which should be observable in reaction norms including a linear part comprised between an upper and a lower plateau (*i.e.* sigmoid shape, Figure 1). Studies on winter metabolic adjustments did highlight linear relationships between temperature and both BMR and Msum but did not test for non-linear effects [21], [23] (but see [22]). Knowledge on the capacity of animals to respond to short-term environmental variability and on thermal thresholds at which endotherms could reach minimal and maximal metabolic values therefore remains limited.

Here, we used the Black-capped chickadee, a small (9–14 g) North-American non-migratory passerine, as our model species to investigate adjustments of BMR and Msum to natural variations in weather parameters. Birds express higher metabolic rates than mammals of comparable size [29] and, given their high surface/volume ratio, small species are highly sensitive to heat loss. This makes the chickadee a perfect model to investigate the effects of weather variability on metabolic flexibility. Chickadees also defend small territories during winter [30], which facilitates recaptures and allows for obtaining sequences of individual measurements in varying conditions. Although temperature undeniably affects avian metabolic rates [21], [31], heat transfer also involves other parameters such as solar radiation, humidity and wind speed [32]–[35]. We therefore considered an array of parameters rather than only the effect of ambient temperature. We expected that, in natural conditions, reaction norms would follow non-linear patterns over the seasonal range of weather variation, as metabolic rates should be limited by physiological constraints [6]. Using random regressions, we tested whether metabolic adjustments differed between individuals in elevation and slope (scenario 1), in elevation only (scenario 2) or neither (scenario 3). To the best of our knowledge, this is the first study to investigate reaction norm of BMR and Msum over natural weather gradients in a free-living population of endotherms.

## Materials and Methods

### (a) Capture and handling

This field study was conducted at the Forêt d'Enseignement et de Recherche Macpès, Québec, Canada (48°18′ N, 68°31′ W) from August 2010 to march 2011. Eighteen capture stations were distributed within the 2300 ha of the forest and were set up with feeders (Perky-Pet 10″ Sunflower Seed and Peanut Feeder) filled with black sunflower seeds. During capture sessions (between 08:00 and 13:00), feeders were removed and homemade potter traps (15 cm×15 cm×15 cm) baited with seeds were used to catch birds, which allowed us to handle individuals as soon as they were trapped. We caught 183 individuals and recaught 45 birds (25%), with an average rate of 1.3±0.2 recapture per bird (table 1). The average duration between two captures was of 45±2 days. Birds caught for the first time were banded with a USGS numbered metal band for identification. After capture, birds were weighed and measured following standardized protocols (length of beak, head plus beak, tarsus, tail and wing) [36]. Following these measurements, up to four birds per day were brought to the field station for metabolic measurements (see [15], [36] for other studies on the same population in which this dataset is also included, for example in multi-year analyses).

**Table 1:** Sample sizes presented relative to the number of capture per bird.

Number of capture/bird	Sample size
1	138
2	27
3	11
4	1
5	3
6	2
7	1

### (b) Ethics statement

All bird manipulations were approved by the animal care committee of the Université du Québec à Rimouski (CPA-37-09-68) and have been conducted under scientific and banding permits from Environment Canada - Canadian Wildlife Service (Permit Number: 10704H).

### (c) Respirometry

At the field station, birds were kept at room temperature in separate cages (39 cm×43 cm×31 cm) supplied with food (sunflower seed) and water *ad libitum* until metabolic rate measurements. Cages were kept in a quiet room receiving natural light. At around 13:00, we measured the Msum of two birds simultaneously using the instruments and protocol described by Petit et al [36]. Measurement of the two remaining birds started before 15:00. Before Msum trials, birds were weighed (±0.1 g) and body temperature (Tb) was measured with a thermocouple reader (Omega model HH-25KC, NIST-traceable, Omega, Montréal, QC, Canada) using a copper-constantan thermocouple inserted into the cloacae approximately 10 mm deep. Then, birds were put in metabolic chambers (effective volume  = 1120 ml) fitted with a perch and a thermistor (Sable Systems UI2 AD converter, Sable Systems, Las Vegas, NV, USA) for chamber temperature measurements. We exposed the birds to helox gas (21% oxygen, 79% helium, average flow rate of 1109 ml.min^−1^) and measured their oxygen consumption (FoxBox oxygen analyzers, Sable Systems, Las Vegas, NV, USA) using a sliding cold exposure protocol [37]. This protocol involved a decrease in ambient temperature of 3°C every 20 minutes with trials starting at 6°C in summer, 3°C in fall and 0°C in winter. Trials ended when birds became hypothermic, which was detectable in real time as a steady decline in oxygen consumption for several minutes. At this time, birds were removed from metabolic chambers and their body mass (Mb) and Tb were measured again. We assumed a bird had reached its Msum when Tb after a trial was ≤38°C (mean Tb before Msum  = 42.35±0.04°C [pers.obs], thus average decline in Tb during Msum measurement>4°C) [38]. Data from birds with Tb above this threshold were discarded (n = 21) (mean Tb of hypothermic birds after Msum  = 34.0±0.2°C). Mb measured before and after trials were averaged and these values were used for Msum analyses. Birds were brought back to their cage with food and water *ad libitum* until BMR measurement commenced at night.

During BMR trials, up to four birds were measured simultaneously from 19:00 to 06:00. Measurements were done at 30°C (within the thermoneutral zone for this species, [39]) using a constant flow of air (average 470 ml.min^−1^). As for Msum, birds were weighed before and after measurements and average Mb were used in BMR analyses.

Oxygen analyzers were adjusted each day to 20.95% O_2_ using CO_2_-free dry air. Mass flow valves (Sierra Instruments, Side-Trak Model 840, Monterey, CA, USA) were calibrated for air and helox using a bubble-O-meter (Dublin, OH, USA) once per year. Metabolic rate calculations were done with ExpeData software, v1.2.6 (Sable Systems, Las Vegas, NV, USA). Using a 20 sec sampling interval for BMR and a 5 sec interval for Msum, BMR and Msum calculations were based respectively on the lowest and highest averaged 10 minutes of oxygen consumption per measurement sequence according to Lighton's equation 10.1 [40]. We applied the instantaneous measurement technique [41] for Msum calculations and a steady state approach for BMR. Duration of BMR trials (around 11 hours) insured that birds were post-absorptive at time of BMR measurement. Since birds use lipids as substrate during fasting and for shivering [12], we estimated energy consumption using a constant equivalent of 19.8 kJ.L^−1^ O_2_ and converted to watts [42]. After BMR measurements, birds were put back in their cage with access to food and water until release on their capture site around 2 hours later.

### (d) Weather data

Body heat loss increases under cold conditions, we therefore considered weather parameters that would most likely affect energy expenditure of small birds during cold exposure. Hence, we used minimal ambient temperature (°C), maximal wind speed (m.s^−1^), minimal absolute humidity (g.m^−3^), minimal solar radiation (W.cm^−2^) and minimal atmospheric pressure (kPa). We chose minimal values for temperature and maximal values for wind speed because a windy cold environment enhances heat loss. We used minimal absolute humidity since dry air facilitates heat loss by evaporation and we employed minimal solar radiation because cloud cover reduces heat gain by radiation. Minimal barometric pressure was considered because storms, which could affect the birds' energy expenditure, are generally preceded by a decrease in atmospheric pressure.

Weather data were recorded during the study by three weather stations (station 1: 48°19′24″ N, 68°31′23″ W, altitude: 166 m; station 2: 48°17′50″ N, 68°31′34″ W, altitude: 176 m; station 3: 48°16′46″ N, 68°33′05″ W, altitude: 188 m) located within the Macpès forest. Each station included instruments at four heights (2 m, 8 m, 14 m and 20 m) that recorded wind speed each 2 minutes and ambient temperature, relative humidity (used to calculate absolute humidity), solar radiation and atmospheric pressure each 15 minutes. For each variable, values from all heights and stations were hourly averaged and these averages were used in our analysis. Unfortunately, equipment failure prevented us from recording accurate solar radiation data.

### (e) Sexing individuals

Of the 183 individual birds captured for this study, 99 (45 females and 54 males) were sexed by PCR analyses (n = 32) [43] or by dissection after metabolic measurements (n = 67) (these birds were sacrificed for a study on organ size flexibility [15]). These birds were then used to establish a discriminant function using morphometric data to determine the sex of the 84 remaining birds (21 females, 29 males, 34 undetermined) using discriminant analyses [44].

### (f) Statistical analysis

To analyse the relationship between metabolic performance and weather variations within the year, we first extracted residual BMR and residual Msum from ANCOVAs that analysed the effects of sex and body mass on whole BMR and Msum. Since minimal ambient temperature and absolute humidity were strongly correlated (r>0.90), we could not include both parameters in models. We therefore used a polynomial regression to extract residual values for absolute humidity controlling for the effect of temperature and included this new variable in further analyses. To characterize the birds' capacity to adjust their BMR and Msum to weather conditions, we investigated variations in residual BMR and residual Msum with random regressions [3], [5] including the four weather parameters (temperature, wind speed, residual absolute humidity and barometric pressure) as fixed effects (inter-correlations r<0.40 in all cases). We also included the individual elevation (*i.e.* individual) and slopes (interaction between individual and each weather variable) as random effects to test for individual effects on metabolic performance adjustments (Figure 1). Hence, for an individual i, the relationship between a phenotype P_i_ and an environmental variable E is defined as: 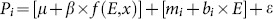
where the fixed effects µ and β are the population mean elevation and slope, the random effects m_i_ and b_i_ are the individual mean elevation and slope and the residual error is ε. f(E,x) is a polynomial function of E elevated to the order x, which allows for determining the shape of the relationship (if x = 0, no relationship; if x = 1, linear; if x>1, nonlinear).

We followed the top-down model selection strategy described by Zuur et al. [45] to determine the best model explaining variations in residual BMR and residual Msum. The procedure goes as follow.

The first step was to determine the structure of the random effects. We used restricted maximum likelihood (REML) estimation to fit several models including the same fixed effects but different random effects. We then compared these models with likelihood ratio tests (LRT) following a chi-square distribution (χ*^2^*) with one degree of freedom. Individual slopes were not significant for residual BMR (p>0.3 in all cases) or residual Msum (p>0.5 in all cases) and were therefore removed from models (*i.e.* scenario 1 was rejected for both metabolic parameters). When individual elevation was significant (*i.e.* data consistent with scenario 2), we calculated repeatability by dividing individual variance by the sum of individual and residual variances. Since multiple measurements on the same individual could potentially influence the data [46], [47], we used LRT to compare random regressions with autoregressive covariance structure to models with unstructured covariance matrix. For both residual BMR and residual Msum, there was no effect of the covariance structure and the correlation estimate was weak (BMR: Χ^2^ = 0.001, p = 0.97, phi = −0.005; Msum: Χ^2^ = 1.27, p = 0.26, phi = 0.24). We therefore used the unstructured covariance matrix in further analyses.

In a second step, we determined the structure of the fixed effects by performing an automated selection based on the corrected Akaike Information Criterion (AICc) of models fitted by maximum likelihood estimation. This allowed us to identify the best models explaining residual BMR and residual Msum variation.

The third step was to use REML estimations to fit the best models and present the following results (full models are presented in tables S1 and S2). To visualize significant effects, we used second order local regressions (loess) with a smoothness parameter of 0.85 to fit curves to the data.

Analyses were performed in R version 3.0.3 [48]. Monthly raw values of BMR and Msum are provided in tables S3 and S4. Data used for the analyses are available in table S5.

## Results

Both BMR and Msum were dependent on sex (BMR: F_2,239_ = 4.6, p<0.05; Msum: F_2,218_ = 3.4, p<0.05) and body mass (BMR: F_1,239_ = 131.4, p<0.0001; Msum: F_1,218_ = 66.0, p<0.0001), with females expressing higher BMR than males (+3.5%, tukey: p<0.01) and higher Msum than individuals of undetermined sex (+6.6%, tukey: p<0.05). Consequently, in subsequent analyses we used the residuals of BMR and Msum, after controlling for sex and body mass.

**Figure 2:**
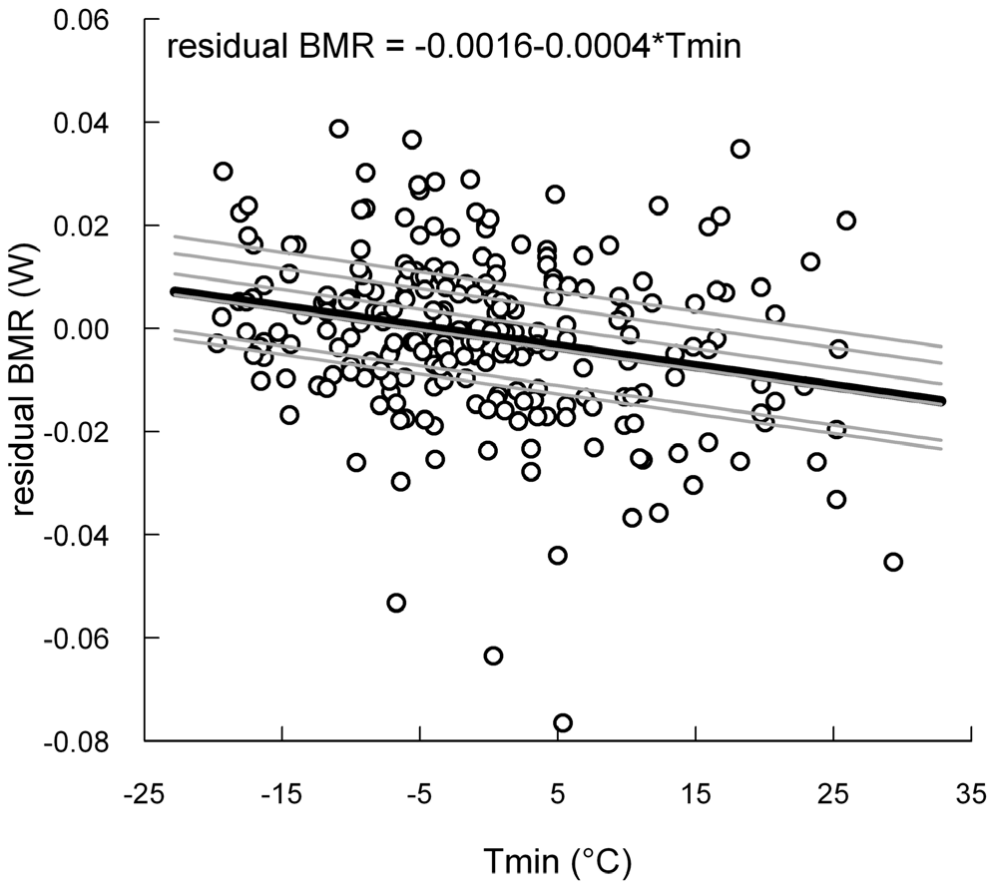
Relationship between mass and sex independent BMR and the natural range of minimal ambient temperature. Residual BMR were extracted from an ANCOVA controlling for body mass and sex. Population-average reaction norm is represented by the black line while grey lines represent reaction norms of individuals caught more than five times.

The best model explaining variations in residual BMR included both minimal ambient temperature (F_1,239_ = 15.8, p<0.0001) and individual elevation (Χ^2^ = 6.9, p<0.01, repeatability  = 0.20). Therefore, BMR reaction norm was consistent with scenario 2. Individual BMR was repeatable and increased with a decline of ambient temperature (Figure 2).

**Figure 3:**
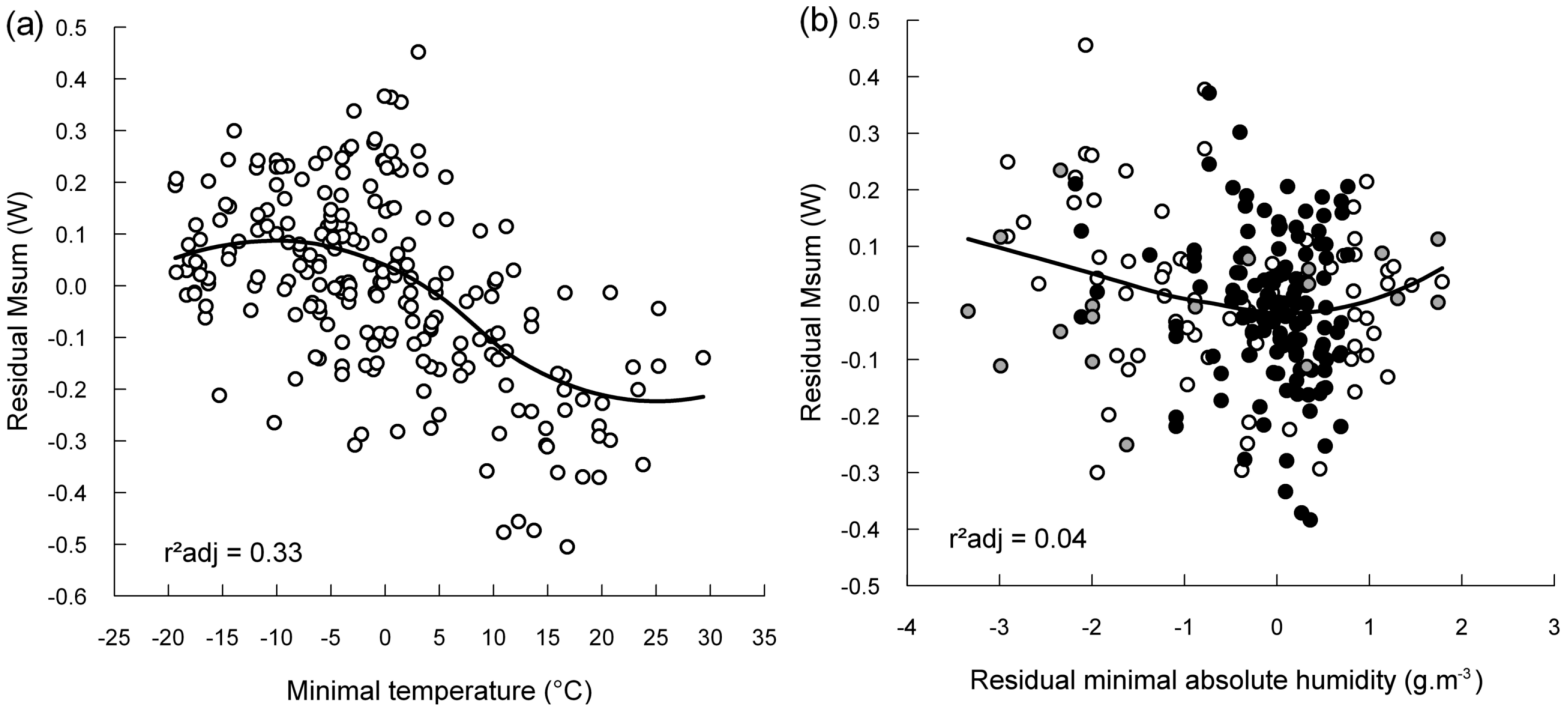
Mass and sex independent Msum variation over the natural range of weather conditions. Data are residual Msum presented against (a) minimal ambient temperature (min Ta) and (b) residual minimal absolute humidity. Black dots in (b) represent data collected at min Ta<0°C, white dots at 0°C<min Ta<16°C and grey dots at min Ta>16°C (see text for details). For both panels, black lines are curves fitted by loess (smoothness  = 0.85, degree  = 2). Residual Msum were extracted from ANCOVA controlling for body mass, sex as well as residual minimal absolute humidity in (a) or minimal temperature in (b). Residual minimal absolute humidity controls for the effect of ambient temperature and was extracted from a polynomial regression. Coefficients of determination are presented for independent relationships, see text for complete model.

Residual Msum did not differ between individuals (p = 0.1) and its reaction norm was consistent with scenario 3. Indeed, residual Msum was best explained by a model (F_6,215_ = 33.3, p<0.0001, r^2^_adj_ = 0.47) including a third order function of both temperature and absolute humidity (Figure 3, table 2). The relationship between residual Msum and minimal temperature (model including only temperature effect: r^2^ = 0.44) followed a Z-shape characterized by a minimal average Msum of 1.23 W reached at 24.0°C and a maximal average Msum of 1.55 W reached at −10°C. Therefore the amplitude of Msum flexibility was of 0.32 W and the slope, calculated in the linear part of the curve (between 2°C and 14°C) was of −0.013 W.°C^−1^ (Figure 3a). The relationship between residual Msum and residuals of minimal absolute humidity followed a U-shape, with the lowest Msum reached at an absolute humidity of 2.8 g.m^−3^ (Figure 3b). However, although the effect was significant, the amount of variation explained by humidity was small since the addition of residual absolute humidity to the model only increased r^2^ by 0.04 (model including temperature and humidity effects: r^2^ = 0.48). Consequently, individual birds expressed similar mass and sex independent Msums, which increased curvilinearly with a decline in ambient temperature and, for a given temperature, residual Msum tended to be higher when the amount of water vapour contained in the air was both low and high within the measured range.

**Table 2:** Best model explaining mass and sex independent Msum variations.

Parameters	Estimate	Standard error	F	p
Intercept	−0.01940	0.01411		
Tmin	−0.01665	0.00177	151.6	<0.0001
Tmin∧2	−0.00041	0.00007	19.2	<0.0001
Tmin∧3	0.00002	0.00001	10.6	<0.01
resAHmin	−0.01382	0.01559	7.8	<0.01
resAHmin∧2	0.04464	0.01390	6.3	<0.05
resAHmin∧3	0.01104	0.00513	4.6	<0.05

Residual minimal absolute humidity was extracted from a polynomial regression and controls for the effect of ambient temperature. resAHmin: residual minimal absolute humidity (g.m^−3^).

## Discussion

Our goal was to investigate reaction norms of BMR and Msum through the natural range of weather variations experienced by a small endotherm. We expected non-linear responses, as metabolic rates would likely be limited by physiological constraints at the extremes of the range [6]. We found that, in Black-capped chickadees, mass and sex independent BMR was explained by variation among individuals and was linearly related to temperature. In contrast, mass and sex independent Msum did not vary among individuals but was related to ambient temperature following a Z-shape relationship, with a linear increase between 24°C and −10°C, and to absolute humidity following a U-shape curve.

### (a) BMR

**Figure 4:**
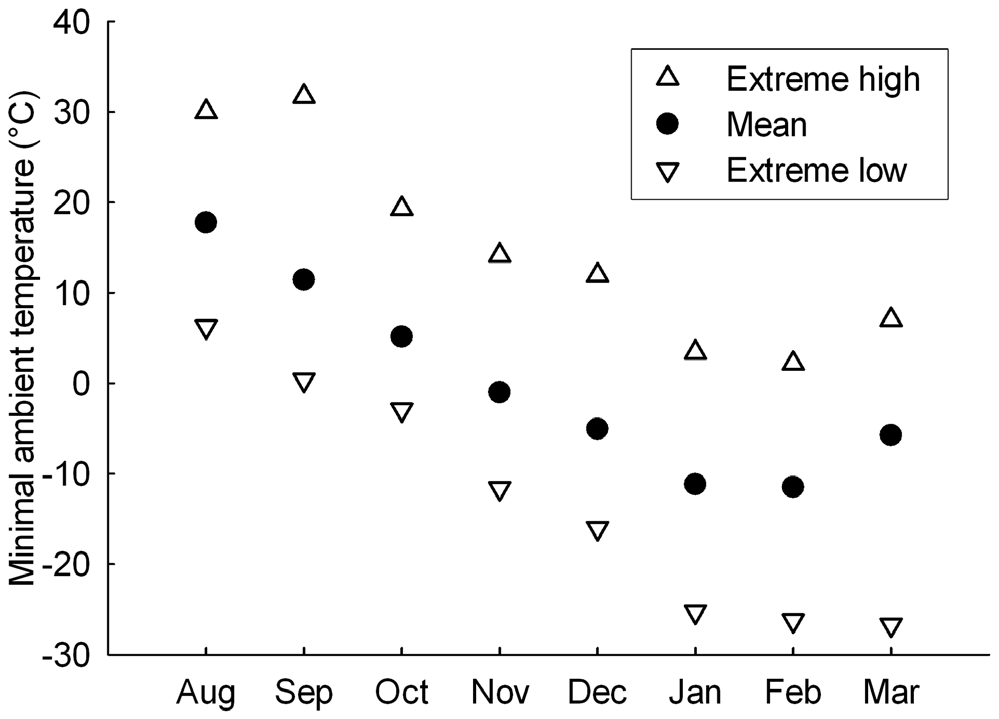
Monthly variations in minimal temperature. Data are extreme low, mean and extreme high values per month for minimal ambient temperature.

Mass and sex independent BMR was negatively correlated with minimal ambient temperature, suggesting that physiological maintenance costs in chickadees increases as ambient temperature decreases, a finding consistent with previous observations in other model species [21], [22]. The relationship was linear over the experienced range of minimal ambient temperatures. This implies that, although the natural variation in temperature was relatively wide (−27°C to +32°C, see Figure 4), BMR did not reach its minimal or maximal limits within this range. This therefore suggests that the range of flexibility in chickadee's BMR is wider than what has been recorded here and previous studies support this interpretation. Indeed, we reported earlier a seasonal increase in chickadee's BMR of only 6% in winter relative to summer for the same year at this location [36] while Sharbaugh [49] and Cooper and Swanson [18] found seasonal differences of 12% and 14% for Alaska and South Dakota populations respectively. Interestingly, at those locations, the natural range of ambient temperature is not much wider (−30°C to +20°C [49] and −10°C to +33°C [18]) than what we reported in this study. Combined with other studies that observed lower BMR in winter relative to summer [50], [51], our results therefore suggest that the constraints driving the BMR reaction norm in Black-capped chickadees is unlikely to result directly from thermoregulatory requirements. Variations in mass and sex independent BMR were also explained by differences among individuals in elevations. Individuals expressed the same rate of change in residual BMR (no effect of individual slopes) and residual BMR was repeatable (R = 0.20) over the measured range of temperature. Therefore, it appears that some individuals consistently maintained a higher BMR relative to others, which suggests that there might be differences among individuals in terms of investment in physiological maintenance (scenario 2), at least in that specific year. Whether this may result in fitness differences among individuals, in which case BMR would reflect individual quality [9], remains to be investigated.

### (b) Msum

Mass and sex independent Msum was related to both minimal temperature and absolute humidity. The correlation between minimal temperature and residual Msum plateaued below -10°C and above 24°C. Between these extremes, chickadees increased their maximal heat production capacity with decreasing ambient temperature. Our temperature data (Figure 4) shows that ambient temperature in August can be as low as 6.3°C while average minimal ambient temperature is already below the 24°C threshold of at this time and location. By October, the warmest minimal temperature never reaches this value. This implies that by the end of August, time at which temperatures are still relatively warm, chickadees are already beginning to improve their thermogenic capacity in preparation for the incoming cold conditions. This interpretation goes in hand with our earlier observations [36] where we demonstrated that Msum in chickadees from this population had already reached 22% of its inter-seasonal cold acclimatized level by October. This finding also suggests, assuming that the norm of reaction for mass and sex independent Msum is constant across populations, that chickadees from the northernmost populations, such as those found in Alaska, may never be able to reduce their thermogenic capacity to its lowest level since maximal summer ambient temperatures typically remains below 20°C at this latitude [49]. These birds may therefore be forced to maintain a certain level of cold acclimatized phenotype even at the warmest time of the year [52].

Chickadees reached their maximal population average residual Msum at a mean minimal temperature of −10°C. This value is well above the lowest temperatures recorded during the coldest months of winter at our site (often <−20°C, see Figure 4) but matches with the average minimal temperature measured at that time (Jan: −11.2°C; Feb: −11.5°C) (Figure 4). Therefore, by the time minimal ambient temperature averaged around −10°C, birds had reached the level of thermogenic capacity that seems to be required for surviving the winter. This suggests that chickadees' thermogenic capacity is adjusted to average environmental conditions rather than to acute temperature extremes.

The highest level of mass and sex independent Msum measured here could represent a physiological and/or a morphological limit [6]. For example, pectoral muscles could be at their maximal size preventing further increases in shivering capacity at temperatures below −10°C [16], [17]. Hence, to face temperature colder than −10°C, birds would have to use strategies such as microhabitat selection [53] or hypothermia [49], [54] to compensate for the lack of endogenous heat production. However, one has to keep in mind that Msum is only an indicator of cold endurance [55] and the level of thermogenic capacity that birds reach when average minimal temperature is around −10°C should be sufficient to support short-term events of temperatures below −10°C. The maximal level of Msum reported here (1.55 W) is similar to what has been observed in Black-capped chickadees from Ohio and Wisconsin (both 1.5 W [56]) but is lower than that reported for South Dakota (2.1 W [56]). Determining to what level Msum can increase above the plateau documented here and the influence of ambient conditions in setting maximal Msum will require further investigation.

Mass and sex independent Msum was also related to residual absolute humidity following a U-shape pattern. For a given ambient temperature, residual Msum was higher when the air contained both relatively low and high amounts of water vapour. Dry air favours evaporation and the loss of body heat [57]. It is therefore not surprising to see that birds tended to maintain a higher thermogenic capacity when conditions were dry for a given temperature. In contrast, finding elevated Msums at high levels of humidity came as a surprise as one would expect the effect of humidity on heat loss to be negligible in the cold due to condensation. However, it is worth noting that 43% of our sample has been collected during days where minimal ambient temperature was above 0°C (Figure 3b) and 35% of our measures were obtained when temperatures were above the freezing point but still below the lower critical temperature for chickadees (16°C in winter and 19.9°C in summer, [18]). It is therefore conceivable that at temperatures above 0°C, water vapour increased heat transfer through air and thus contributed to increase body heat loss and, consequently, individual's maximal thermogenic capacity. We nevertheless must emphasize that the number of observations at high levels of humidity, where residual Msum was found to increase, were relatively few and contained measurements on days where temperatures were above the lower critical temperature (Figure 3b). Although the cooling effect of water vapour at these temperatures was apparently sufficient to trigger an upregulation of Msum, this interpretation must nonetheless be considered with caution and requires further investigations.

Residual Msum varied with temperature and humidity but did not differ among individual birds. Therefore, for a given mass and sex, individual chickadees expressed similar variations in Msum throughout the year (scenario 3). As maximal thermogenic capacity is thought to reflect long-term cold endurance [55] and survival [23], this suggests that Msum could be under stabilizing selection pressure, which would work to eliminate extreme phenotypes. Theoretically, individuals with low levels of Msum may not be able to survive episodes of prolonged cold spells while constantly maintaining a higher capacity than required might reduce available resources for other fitness-related physiological functions such as immunity [58] or defence against oxidative damage [59]. Such stabilizing selection would lead to a low degree of individual variation in Msum as we observed here, although, this may only be true for specific sets of winter conditions as recent evidences suggest that repeatability of Msum may be year-specific [60]). Alternatively, while following the general Z-shape pattern, residual Msum could also be highly variable within individuals, which would prevent us from detecting any Msum consistency and therefore from highlighting between-individual differences.

To the best of our knowledge, despite a relatively small recapture sample size (see [61] for suggested requirements with random regressions), this study is the first to investigate reaction norms of physiological parameters (*i.e.* metabolic performance) over the natural range of weather conditions in small free-living endotherms, both at the individual and population levels. We demonstrated that physiological maintenance costs were linearly related with ambient temperature but not limited within the measured range and were individual-dependent in our population. In contrast, heat production capacity, and thus cold tolerance, was not dependent on individuals but was related to both ambient temperature and absolute humidity following cubic relationships potentially highlighting limits to physiological adjustments. This supports the assertion that basal and maximal cold-induced metabolic rates are functionally uncoupled and that phenotypic flexibility of these traits may be responding to different and possibly independent constraints [36].

## Supporting Information

Table S1 **Full model explaining mass and sex independent BMR variation.** Residual BMR were extracted from an ANCOVA controlling for body mass and sex. Residual minimal absolute humidity was extracted from a polynomial regression and controls for the effect of ambient temperature.
(XLS)

Table S2 **Full model explaining mass and sex independent Msum variation.** Residual Msum were extracted from an ANCOVA controlling for body mass and sex. Residual minimal absolute humidity was extracted from a polynomial regression and controls for the effect of ambient temperature.
(XLS)

Table S3 **BMR monthly variation.** Least square means of BMR were calculated from a linear mixed effect model including body mass and month as fixed parameters and bird ID as random variable.
(XLS)

Table S4 **Msum monthly variation.** Least square means of Msum were calculated from an ANCOVA including body mass and month as fixed parameters.
(XLS)

Table S5 **Data used for the analyses.**
(XLS)
